# DNA intercalator stimulates influenza transcription and virus replication

**DOI:** 10.1186/1743-422X-8-120

**Published:** 2011-03-15

**Authors:** Olive TW Li, Leo LM Poon

**Affiliations:** 1 State Key Laboratory for Emerging Infectious Diseases, Department of Microbiology and the Research Centre of Infection and Immunology, The University of Hong Kong, Hong Kong

## Abstract

Influenza A virus uses its host transcription machinery to facilitate viral RNA synthesis, an event that is associated with cellular RNA polymerase II (RNAPII). In this study, various RNAPII transcription inhibitors were used to investigate the effect of RNAPII phosphorylation status on viral RNA transcription. A low concentration of DNA intercalators, such as actinomycin D (ActD), was found to stimulate viral polymerase activity and virus replication. This effect was not observed in cells treated with RNAPII kinase inhibitors. In addition, the loss of RNAPII_a _in infected cells was due to the shift of nonphosphorylated RNAPII (RNAPII_a_) to hyperphosphorylated RNAPII (RNAPII_o_).

## Introduction

The C-terminal domain (CTD) of RNAPII is important for cellular mRNA transcription, and interacts with several post-transcriptional factors for RNA maturation and nuclear export. The phosphorylation status of CTD is known to be a critical regulatory checkpoint for RNAPII transcription [1]. The hyperphosphorylated (transcriptionally engaged) form of RNAPII is designated as RNAPII_o_, whereas its nonphosphorylated (transcriptionally inactive) form is designated as RNAPII_a_. At the early stage of transcription, free RNAPII_a _interacts with other general transcription factors on cellular DNA promoters to form a transcription pre-initiation complex, which is followed by transcription initiation [2]. The newly initiated RNAPII_a _then proceeds to the promoter-proximal pause region, and the paused RNAPII_a _is subsequently hyperphosphorylated, preferably on the serine 5 (Ser5) positions, by cyclin-dependent kinase (Cdk) 7. As transcription elongation proceeds, the serine 2 (Ser2) and Ser5 positions in the CTD of RNAPII are hyperphosphorylated by Cdk9 [3] and dephosphorylated by SCP1 [4], respectively. The Ser5-phosphorylation helps to recruit enzymes to cap the nascent RNA transcript, whereas the Ser2-phosphorylation facilitates the conversion of RNAPII into a productive elongating form.

Influenza viral RNA synthesis is dependent on its host transcription machinery. Various RNAPII inhibitors such as α-amantin and actinomycin D (ActD) have been shown to inhibit influenza virus replication [5-7]. Chan *et al. *demonstrated that the influenza viral polymerase complex can inhibit RNAPII transcription elongation, but not initiation [8], a phenomenon that is similar to the transcriptional arrest of RNAPII. This transcriptional arrest may be related to direct interaction between vRNP and Ser5-phosphorylated RNAPII_o _[9]. It has also been demonstrated that a robust polymerase complex is more capable of binding to RNAPII_o _[10]. Recently, influenza viral polymerase has been proposed to induce the direct degradation of RNAPII_a _[11-13], thereby inhibiting host gene expression. The overall conclusion of these previous findings is that RNAPII plays a critical role in viral RNA transcription, although little is known about the mechanism responsible for RNAPII_a _disappearance during infection. Moreover, the role played by the post-translation modification of RNAPII in viral RNA synthesis is yet to be determined. In this study, we would like to determine the effect of various RNAPII inhibitors on influenza viral polymerase functions and virus replications. In particular, the inhibitors used in this study are known to inhibit RNAPII via different mechanisms and have different effects on the phosphorylation status of RNAPII. It is of our interest to use these chemicals to understand how the influenza virus can utilize RNAPII to facilitate viral RNA synthesis.

## Findings

**Figure 1:**
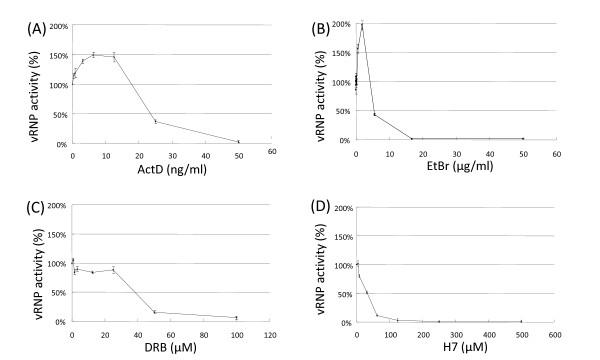
**Effects of RNAPII transcription inhibitors on influenza viral polymerase activity**. 293T cells were transfected with A/WSN/33 PB2, PB1, PA and NP protein expression plasmids and a pPolI Luc-RT RNA expression plasmid, as described previously [10]. The transfected cells were then washed and replenished with media containing various concentrations of ActD (A), DRB (B), H7 (C) or EtBr (D) at six hours post-transfection. The luciferase activity of the drug-treated cells was measured with a luminometer (Victor3, PerkinElmer) using a Steady-Glo luciferase reagent (Promega) at 22 hours post-transfection. The luciferase activity of the mock-treated cells was taken as 100% polymerase activity. Data ± SE were obtained from the triplicate experiments.

This study examined the effects of various RNAPII transcription inhibitors on viral RNA synthesis. A luciferase-based influenza viral polymerase reporter assay [10] was used to measure the viral polymerase activity in drug-treated cells. Transfected cells were first treated with different RNAPII inhibitors at six hours post-transfection and then tested for luciferase activity at 22 hours post-transfection (Figure 1). ActD, a DNA intercalator that is well-known to convert RNAII_a _to RNAPII_o _[14], was found to inhibit viral polymerase activity at high concentrations (Figure 1A). Strikingly, however, ActD at the low concentration range (~10 ng/ml) was consistently found to stimulate viral polymerase activity by 50%. This ActD activation effect was previously observed in genes containing an HIV-1 LTR sequence [15]. ActD at this low concentration range can increase the RNAPII_o _population by creating temporary transcriptional obstacles for RNAPII_o _[15,16], which suggests that the blockage of RNAPII_o _transcription may facilitate viral gene expression. This activation effect was further confirmed by the use of another DNA intercalator, ethidium bromide (EtBr), to induce the stalling of RNAPII_o_. As shown in Figure 1B, a two-fold increase in viral polymerase activity was observed in cells treated with 2.5 μg/ml of EtBr. In contrast, Cdk inhibitors 5,6-dichlorobenzimidazole riboside (DRB) and 1-(5'-isoquinolinesulfonyl)-2-methylpiperazine (H7), which can inhibit the phosphorylation of RNAPII_a_, failed to exhibit similar stimulating effects on such activity (Figures 1C and 1D). Using a GFP expression plasmid under the control of a CMV promoter as a control, it was then confirmed that these DNA intercalators in the concentrations under investigation cannot enhance cellular RNAPII transcription [15] (Additional File 1). In short, these results suggest that influenza viral polymerase may require RNAPII_o_, or the formation of RNAPII_o_, for efficient viral transcription.

**Figure 2:**
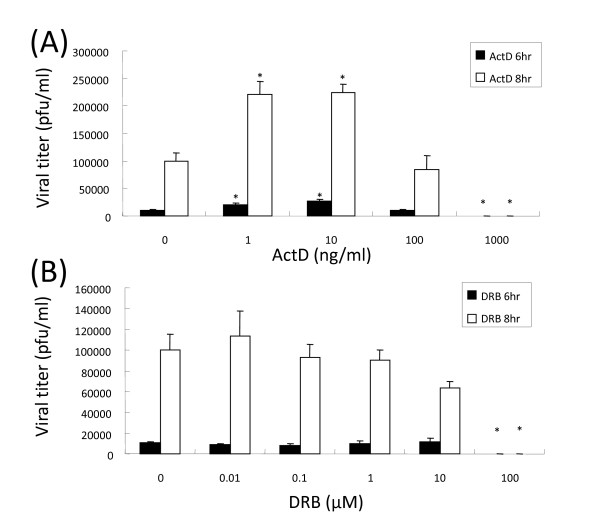
**Actinomycin D stimulates virus replication at early post-infection stage**. MDCK cells were infected with A/WSN/33 at an MOI of 10 for one hour, followed by a short 0.9% saline (pH 2.0) wash. The infected cells were then cultured in media supplemented with various concentrations of ActD (A) or DRB (B). Viral culture supernatants were harvested at the indicated time points, and viral titres were titrated using standard plaque assay techniques. Data ± SE were obtained from the triplicate experiments. Asterisk: viral titres that were significantly different from their corresponding controls (p < 0.05).

To test whether the unexpected enhancement effect of ActD on viral polymerase has any impact on virus replication, MDCK cells were treated with various concentrations of ActD immediately after viral infection. Briefly, MDCK cells were infected with A/WSN/33 for one hour. In order to observe the maximal effect of this drug on a single round of virus replication, cells were super-infected with the virus at an MOI of 10. The uninfected virus in the inoculums was inactivated by a short acidic buffer wash after infection. The amount of progeny viral particles generated from the treated cells at six (i.e. <1 virus replication) and eight (i.e. ~1 virus replication cycle) hours post-infection was determined. As shown in Figure 2A, influenza virus replication can be abolished by treating infected cells with 1 μg/ml of ActD, as expected [17]. Unlike the results obtained from the transfected 293T cells used in the aforementioned luciferase assay, MDCK cells treated with 100 ng/ml of ActD can still support virus replication. These observations suggested these cell lines might have different tolerances to the drug. Nonetheless, a low concentration of ActD was also found to enhance virus replication significantly (p < 0.05). For example, the viral titre from cells treated with 1 ng/ml of ActD at 8 hours post-infection was found to be 2.2-fold higher than that of the mock control. This stimulating effect, however, was not observed in cells treated with DRB (Figure 2B). These results indicate that different RNAPII inhibitors may have different effects on virus replication and that these differential effects may be due to different phosphorylation statuses of CTD (see below).

**Figure 3:**
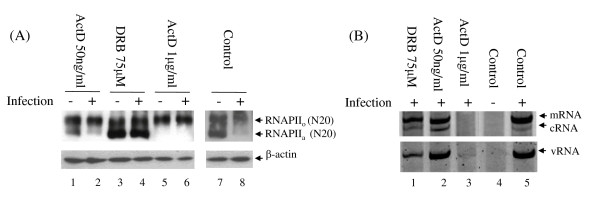
**Effects of ActD and DRB on RNAPII phosphorylation and viral transcription**. Prior to infection, MDCK cells were treated with ActD or DRB in MEM supplemented with 10% foetal calf serum and 1% penicillin/streptomycin for three hours, followed by infection with A/WSN/33 (MOI = 2) in the presence of the corresponding drugs. The infected cells were cultured in a medium containing ActD or DRB, and culture supernatants were harvested at six hours post-infection. (A) Effects of ActD and DRB on RNAPII phosphorylation in infected cells. Total cell lysates were harvested and studied by western blot analysis using anti-RNAPII (N20) and anti-β actin (C4) antibodies (Santa Cruz Biotechnology, Santa Cruz, USA). The signals for RNAPII_o _and RNAPII_a _are indicated. Beta-actin was used as the loading control. (B) Effects of ActD and DRB on viral RNA synthesis. Total RNA was analyzed with primer extension assays, as described previously (10). The bands representing the mRNA, cRNA and vRNA of segment 5 are marked by arrows.

ActD intercalates DNA, and inhibits transcription elongation by immobilizing the RNAIIP_o _on DNA templates [18]. DRB, in contrast, is a Cdk inhibitor that inhibits the phosphorylation of RNAPII_a _[19]. We therefore took advantage of the distinct inhibitory mechanisms of these two chemicals to investigate the disappearance of RNAPII_a _in influenza virus-infected cells. MDCK cells were pre-incubated with ActD or DRB at a predetermined concentration known to have prominent change on CTD phosphorylation in the Western blot analyses (Figure 3A, lanes 1- 4), but without severely affecting the viral RNA transcription and replication in the subsequent viral infection (Figure 3B, lanes 2 and 1). As shown in Figure 3A (lane 8), influenza viral infection promoted the disappearance of RNAPII_a _in the untreated cells as described in previous investigations [10,11,13]. Cells treated with 1 μg/ml of ActD had a complete conversion of RNAPII_a _to RNAPII_o _(Figure 3A, lanes 5 and 6), but failed to support viral RNA synthesis ( 3B, lane 3). Cells treated with 50 ng/ml of ActD had reduced levels of RNAPII_a _(Figure 3A, lane 1) and remained able to support viral RNA synthesis (Figure 3B, lane 2). It should be noted that viral infection is still capable of inducing the disappearance RNAPII_a _at this concentration of ActD (Figure 3A; compare lanes 1 and 2), suggesting that the phophorylation of RNAPII_a _is essential for virus replication. On the other hand, viral transcription and replication products were detected in the infected cells treated with 75 μM of DRB (Figure 3B, lane 1), although the treatment was found to inhibit the disappearance of RNAPII_a _induced by the infection (Figure 3A, lane 4). Previous co-immunoprecitation work has demonstrated that viral polymerase interacts specifically with Ser5-phosphorylated, but not Ser2-phophorylated, RNAPII_o _[9]. Hence, the results of both the current and previous studies suggest that viral polymerase may need to recruit and arrest newly formed RNAPII_o _(i.e., Ser-5-phophorylated RNAPII), but not the actively elongating form of RNAPII_o _(i.e., Ser-2-phophorylated RNAPII), for viral RNA transcription.

## Conclusion

Influenza A virus infection results in a significant loss of transcriptionally inactive RNAPII (RNAPII_a_) [11-13]. However, as influenza polymerase requires capped primers snatched from the host nuclear RNA for its viral RNA transcription [20-22], a direct induction of RNAPII_a _degradation via the viral polymerase may not favour such a transcription. In this study, it has been demonstrated here that the disappearance of RNAPII_a _is related to a shift of RNAPII_a _to RNAPII_o _(Figure 3). In addition, this conversion of RNAPII_a _to RNAPII_o _is found to be important to viral RNA synthesis, which suggests that newly synthesized RNAPII_o _may be a critical determinant of viral transcription. RNAPII can be subjected to various post-translational modifications [1]. Further investigation of the post-translational modification of RNAPII in influenza virus-infected cells may help us to better understand the transcription and replication of influenza viruses.

## Competing interests

The authors declare that they have no competing interests.

## Authors' contributions

OTWL designed the study and conducted the experiments. LLMP supervised the project. Both OTWL and LLMP analysed the data, wrote the manuscript and approved the final version of the manuscript.

## Supplementary Material

Additional file 1 **Effects of EtBr on GFP expression**. 293T cells were transfected with GFP expressing plasmid under the control of a CMV promoter. The transfected cells were then washed and replenished with media containing various concentrations of EtBr at six hours post-transfection. The GFP signal was measured with a luminometer (Victor3, PerkinElmer) at 22 hours post-transfection. The GFP signal of the mock-treated cells was taken as 100% polymerase activity. Data ± SE were obtained from the triplicate experiments. Click here for file
