# Supplementary material for: DNA intercalator stimulates influenza transcription and virus replication
Source: Virol J. 2011 Mar 15;8:120. doi: 10.1186/1743-422X-8-120 (PMC3069954; doi:10.1186/1743-422X-8-120)
Supplement: Additional file 1 — Effects of EtBr on GFP expression. 293T cells were transfected with GFP expressing plasmid under the control of a CMV promoter. The transfected cells were then washed and replenished with media containing various concentrations of EtBr at six hours post-transfection. The GFP signal was measured with a luminometer (Victor3, PerkinElmer) at 22 hours post-transfection. The GFP signal of the mock-treated cells was taken as 100% polymerase activity. Data ± SE were obtained from the triplicate experiments. [file 1743-422X-8-120-S1.PPT]

## Slide 1
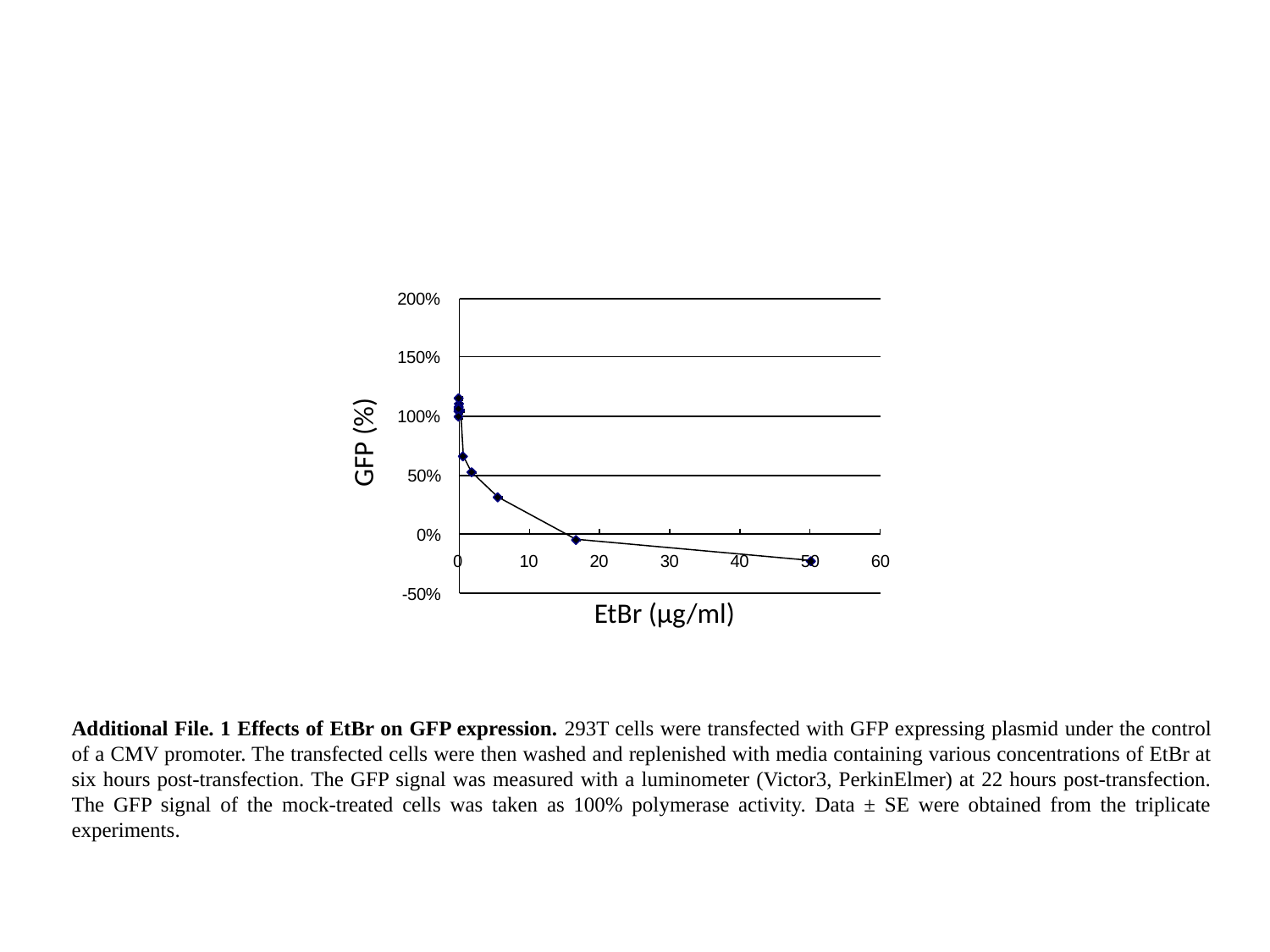

GFP (%)
EtBr (μg/ml)
Additional File. 1 Effects of EtBr on GFP expression. 293T cells were transfected with GFP expressing plasmid under the control of a CMV promoter. The transfected cells were then washed and replenished with media containing various concentrations of EtBr at six hours post-transfection. The GFP signal was measured with a luminometer (Victor3, PerkinElmer) at 22 hours post-transfection. The GFP signal of the mock-treated cells was taken as 100% polymerase activity. Data ± SE were obtained from the triplicate experiments.
